# Differences in prognostic relevance of rectal magnetic resonance imaging findings before and after neoadjuvant chemoradiotherapy

**DOI:** 10.1038/s41598-019-46499-9

**Published:** 2019-07-11

**Authors:** Kwang-Seop Song, Dong Woon Lee, Bun Kim, Bo Yun Hur, Min Jung Kim, Min Ju Kim, Chang Won Hong, Sung Chan Park, Hyoung-Chul Park, Dae Kyung Sohn, Byung Chang Kim, Kyung Su Han, Jae Hwan Oh

**Affiliations:** 1 Research Institute and Hospital, National Cancer Centre, Centre for Colorectal Cancer, Goyang, 10408 Korea; 2 Seoul National University College of Medicine, Healthcare System Gangnam Centre, Department of Radiology, Seoul, 06236 Korea; 3 Seoul National University College of Medicine, Seoul National University Hospital, Department of Surgery, Seoul, 03080 Korea; 4 Research Institute and Hospital, National Cancer Centre, Department of Radiology, Goyang, 10408 Korea

**Keywords:** Cancer imaging, Rectal cancer

## Abstract

This retrospective study was designed to compare prognostic relevance of magnetic resonance imaging (MRI) findings before and after neoadjuvant chemoradiotherapy (CRT). From 2002 to 2010, 399 patients who underwent surgery after CRT for rectal cancer (≥T3) and had adequate pre-CRT (mr) and post-CRT (ymr) MRI findings were examined. Factors examined included tumour (T), lymph node (N), mesorectal fascia (MRF), extramural venous invasion (EMVI), and tumour regression grade (TRG). Two Cox proportional hazard models were created using mr and ymr findings separately for overall survival (OS), disease-free survival (DFS), and local recurrence rate (LRR). Among mr findings, only mrEMVI was a significant prognostic factor for OS and DFS. Among ymr findings, ymrN, ymrMRF, and ymrEMVI were significant prognostic factors for OS and DFS, whereas ymrMRF and ymrEMVI were significant prognostic factors for LRR. C-indices tended to be higher for ymr findings than for mr findings (OS, 0.682 vs. 0.635; DFS, 0.660 vs. 0.631; LRR, 0.701 vs. 0.617). Survival outcomes of patients having all ymr risk factors were significantly poor (5-year OS, 52.4%; 5-year DFS, 38.1%; 5-year LRR, 27.7%). ymr findings showed better prognostic significance than mr findings. Among ymr findings, ymrN, ymrMRF, and ymrEMVI were independent prognostic factors for oncologic outcomes.

## Introduction

Rectal magnetic resonance imaging (MRI) plays an important role in determining rectal cancer treatment. Practical guidelines recommend neoadjuvant therapy according to the initial clinical stage of rectal cancer, and MRI is one of the reliable tools for initial staging^1^. Additionally, several studies have used MRI for assessing the response of rectal cancer to neoadjuvant therapy and have attempted personalised treatments such as local excision or induction chemotherapy^2,3^.

Various MRI findings of tumour (T), lymph node (N), mesorectal fascia (MRF), extramural venous invasion (EMVI), and tumour regression grade (TRG) are based on pathologic findings and known to be related to the prognosis of patients with rectal cancer^4^. The postoperative T and N classifications are one of the traditional prognostic factors that have been used for a long period of time. The T and N staging of MRI have been used under the same context. Moreover, pathologic T and N classifications after neoadjuvant chemoradiotherapy (CRT) have been reported to represent the prognosis of patients^5^. However, the accuracy and prognostic significance of MRI restaging are controversial due to tissue changes such as fibrosis and oedema^4^.

The MRF is a factor in determining the circumferential resection margin (CRM) during total mesorectal excision^6^. Adam *et al*. have reported that postoperative CRM is an important prognostic factor in rectal cancer surgery^7^. MRI can accurately evaluate MRF involvement in rectal cancer^8^, and the MERCURY group reported that MRF involvement on MRI is associated with poor disease-free survival (DFS) and local recurrence rate (LRR)^8,9^.

EMVI, defined as the presence of tumour cells in blood vessels outside the muscular layer, has been also known as a poor prognostic factor^10^. Smith *et al*. developed a scoring system to evaluate EMVI through high-resolution MRI^11^. Thereafter, several studies have demonstrated that grade 3 and 4 EMVI findings on MRI are associated with poor prognosis^11–14^.

Pathologic TRG is an indicator of rectal cancer response to CRT, and patients with less regression have poor prognosis^15^. The MERCURY group applied a similar concept into MRI and showed that TRG on MRI after CRT could be a prognostic factor^9^.

Tissue changes after CRT could affect the accuracy and prognostic significance of MRI findings^4^. However, little is known whether the prognostic significance of MRI findings differ before and after CRT, and it is unclear which point of assessment is more appropriate for representing prognosis. Only few studies have evaluated the differences in prognostic significance of MRI findings before and after CRT^12,14,16^. Furthermore, it is also uncertain that these MRI findings are independent prognostic factors for each other because most of the previous studies did not include all  MRI findings^9,12,14^. Thus, this study aimed to determine the prognostic significance of pre-CRT MRI (mr) and post-CRT MRI (ymr) findings in patients with rectal cancer.

## Results

### Patient and MRI characteristics with survival outcomes

The median follow-up periods for OS, DFS, and LRR were 103 (range, 12–181), 74 (range, 0–172), and 81 (range 0–174) months, respectively. Distant metastasis (DM) occurred in 88 patients during the follow-up period. Local recurrence developed in 29 patients and was accompanied by DM in 24 patients. Nineteen patients experienced secondary malignancy in other organs, and secondary primary colon cancer occurred in two patients. At the end of the observation, a total of 102 patients died.

Table 1 summarizes the survival outcomes according to patient and MRI characteristics. Compared to the mr findings, most of the ymr findings were significant for survival outcome. Similarly, many variables such as T, N, MRF, and EMVI in downstaging after CRT were also significantly related to better prognosis (Supplementary Table 1). Validity and reliability assessment between MR findings and pathology were summarised in Supplementary Table 2.

**Table 1:** Patient and tumour characteristics with survival outcome.

	No. of Pts.	Overall survival			Disease free survival			Local recurrence rate		
		5y OS	95% CI	*P*	5y DFS	95% CI	*P*	5y LRR	95% CI	*p*
Sex				0.231			**0.042**			**0.019**
Male	265	86.0	82.0–90.3		71.8	66.4–77.6		9.1	5.4–12.7	
Female	134	88.8	83.6–94.3		80.0	73.4–87.2		1.7	0.0–4.0	
Age				**0.001**			0.330			0.435
<65	289	88.2	84.6–92.0		75.3	70.5–80.5		7.3	4.1–10.3	
≥65	110	83.6	77.0–90.8		72.5	64.1–82.0		4.5	0.0–8.7	
Location				0.065			**0.029**			0.094
>5 cm	274	89.1	85.4–92.8		77.4	72.5–82.7		4.8	3.1–7.4	
≤5 cm	125	82.4	76.0–89.4		68.3	60.4–77.1		10.6	4.7–16.1	
CEA				**0.003**			**0.008**			0.102
≤5 ng/ml	259	88.0	84.2–92.1		77.8	72.7–83.1		6.0	2.9–9.1	
>5 ng/ml	140	85.0	79.3–91.1		68.7	61.2–77.0		7.6	3.0–12.0	
mrT stage				0.772			0.102			0.153
T3	363	87.1	83.7–90.6		76.0	71.6–80.6		5.8	3.2–8.3	
T4	36	86.1	75.5–98.2		61.0	46.9–79.2		14.2	1.9–25.0	
mrN stage				0.242			0.108			0.120
N0	89	92.1	86.7–97.9		81.2	73.0–90.3		2.5	0.0–5.9	
N1/N2	189/121	85.5	81.7–89.5		72.8	67.9–78.0		7.7	4.6–10.8	
mrMRF				0.384			0.054			0.097
(−)	300	89.0	85.5–92.6		77.9	73.2–82.9		4.9	2.3–7.5	
(+)	99	80.8	73.4–89.0		64.6	55.7–74.9		11.8	5.0–18.1	
mrEMVI				**0.026**			**0.002**			0.145
(−)	207	91.8	88.1–95.6		80.7	75.3–86.4		5.3	2.0–8.4	
(+)	192	81.8	76.5–87.4		67.9	61.5–75.0		8.0	3.9–12.0	
ymrT				0.065			**0.010**			0.253
T0/T1/T2	17/5/107	90.7	85.8–95.9		81.9	75.3–89.0		5.1	1.0–8.9	
T3/T4	239/31	85.2	81.1–89.5		71.0	65.7–76.8		7.4	4.0–10.6	
ymrN				** < 0.001**			** < 0.001**			**0.036**
N0	267	91.4	88.1–94.8		80.6	75.9–85.6		5.3	2.4–8.1	
N1/N2	96/36	78.0	71.3–85.4		62.5	54.6–71.5		9.3	3.9–14.4	
ymrMRF				**0.010**			**0.002**			**0.006**
(−)	340	88.8	85.5–92.2		77.4	73.0–82.1		4.9	2.5–7.3	
(+)	59	76.3	66.2–87.9		57.9	46.4–72.3		16.6	6.1–26.0	
ymrEMVI				** < 0.001**			** < 0.001**			**0.008**
(−)	302	90.7	87.5–94.1		79.3	74.7–84.2		5.5	2.8–8.2	
(+)	97	75.3	67.1–84.4		60.1	51.0–70.8		9.9	3.5–15.8	
mrTRG				** < 0.001**			**0.001**			**0.007**
G1/G2/G3	11/79/258	89.7	86.5–92.9		77.2	72.8–81.8		5.3	2.8–7.7	
G4/G5	49/2	68.6	57.0–82.6		56.3	43.8–72.5		17.2	4.5–28.2	

5 y; 5-year, OS; overall survival, DFS; disease free survival, LRR; local recurrence rate, No.; number, Pts.; patients, CI; confidence interval, CEA; carcinoembryonic antigen, mr; magnetic resonance imaging before neoadjuvant chemoradiotherapy, T; tumour stage, N; lymph node stage, MRF; mesorectal fascia involvement, EMVI; extramural venous invasion, ymr; magnetic resonance imaging after neoadjuvant chemoradiotherapy, mrTRG; magnetic resonance tumour regression grade, G; grade.

### Harrell’s C-index for each MRI finding with adjustment for clinical variables

In the analysis of OS, Harrell’s C-indices for EMVI were the highest among mr and ymr findings, being 0.656 and 0.680, respectively. For DFS, EMVI had the highest C-index (0.634) among mr findings, while N had the highest C-index (0.661) among ymr findings. For LRR, EMVI also showed the highest C-indices among mr and ymr findings, being 0.720 and 0.728, respectively (Table 2).

**Table 2:** C-index of each MRI finding for survival outcome.

Variable [reference]	Overall survival	Disease free survival	Local recurrence rate
mrT4 [mrT3]	0.627 (0.568–0.686)	0.605 (0.550–0.660)	0.704 (0.596–0.812)
mrN(+) [mrN(−)]	0.637 (0.578–0.696)	0.609 (0.554–0.664)	0.715 (0.607–0.823)
mrMRF(+) [mrMRF(−)]	0.629 (0.570–0.688)	0.613 (0.558–0.668)	0.716 (0.608–0.824)
mrEMVI(+) [mrEMVI(−)]	**0.656 (0.597–0.715)**	**0.634 (0.579–0.689)**	**0.720 (0.612–0.828)**
ymrT3–4 [ymrT0–2]	0.636 (0.577–0.695)	0.621 (0.566–0.676)	0.708 (0.600–0.816)
ymrN(+) [ymrN(−)]	0.672 (0.613–0.731)	**0.661 (0.606–0.716)**	0.717 (0.609–0.825)
ymrMRF(+) [ymrMRF(−)]	0.637 (0.578–0.696)	0.617 (0.562–0.672)	0.707 (0.599–0.815)
ymrEMVI(+) [ymrEMVI(−)]	**0.680 (0.621–0.739)**	0.647 (0.592–0.702)	**0.728 (0.620–0.836)**
mrTRG G4–5 [mrTRG G1–3]	0.652 (0.593–0.711)	0.622 (0.567–0.677)	0.722 (0.614–0.830)

Age, sex, location and CEA were adjusted.

Bold numbers indicate the highest c-index among pre- and post-CRT MRI findings for each survival outcome.

mr; magnetic resonance imaging before neoadjuvant chemoradiotherapy, ymr; magnetic resonance imaging after neoadjuvant chemoradiotherapy, T; tumour stage, N; lymph node stage, MRF; mesorectal fascia involvement, EMVI; extramural venous invasion, mrTRG; magnetic resonance tumour regression grade, G; grade, CRT; neoadjuvant chemoradiotherapy.

### Multivariable analysis

The multivariable analysis of OS, DFS, and LRR was performed using mr and ymr findings separately.

The multivariable analysis of OS showed that only mrEMVI remained significant among mr findings (hazard ratio [HR], 1.54; p = 0.033). Among ymr findings, ymrN, ymrMRF, and ymrEMVI were significant variables, having HRs of 1.63 (p = 0.037), 1.76 (p = 0.023), and 1.73 (p = 0.029), respectively. Regarding mr and ymr findings for OS, Harrell’s C-indices for the final models were 0.635 (95% confidence interval [CI], 0.576–0.694) and 0.682 (95% CI, 0.623–0.741), respectively (Table 3).

**Table 3:** Multivariable analysis of pre- and post-CRT MRI findings.

Variable [reference]	VIF	OS (event = 102)			DFS (event = 116)			LRR (event = 29)		
		HR	95% CI	P	HR	95% CI	P	HR	95% CI	p
Female [male]	1.01				0.68	0.45–1.02	0.064	0.30	0.11–0.87	0.027
Age^a^	1.03	1.04	1.02–1.06	<0.001						
Location <5 cm [≥5 cm]	1.16				1.87	1.26–2.78	0.002			
CEA>5 ng/ml [≤5 ng/ml]	1.05	1.67	1.13–2.47	0.011	1.42	0.98–2.05	0.068			
mrT4 [mrT3]	1.44									
mrN(+) [mrN(−)]	1.15									
mrMRF(+) [mrMRF(−)]	1.51									
mrEMVI(+) [mrEMVI(−)]	1.24	1.54	1.04–2.29	0.033	2.05	1.38–3.04	<0.001			
C index of final model		0.635 (0.576–0.694)			0.631 (0.576–0.686)			0.617 (0.529–0.705)		
Female [male]	1.02				0.61	0.40–0.92	0.019	0.29	0.10–0.83	0.021
Age^a^	1.04	1.04	1.02–1.06	<0.001						
Location<5 cm [≥5 cm]	1.08				1.56	1.05–2.30	0.027			
CEA>5 ng/ml [≤5 ng/ml]	1.07	1.45	0.97–2.17	0.073	1.43	0.98–2.07	0.061			
ymrT3–4 [ymrT0–2]	1.29									
ymrN(+) [ymrN(−)]	1.40	1.63	1.03–2.58	0.037	1.84	1.21–2.80	0.004			
ymrMRF(+) [ymrMRF(−)]	1.27	1.76	1.08–2.86	0.023	1.56	0.99–2.46	0.054	2.72	1.23–6.02	0.013
ymrEMVI(+) [ymrEMVI(−)]	1.53	1.73	1.06–2.83	0.029	1.67	1.07–2.63	0.026	2.42	1.14–5.11	0.021
mrTRG G4–5 [mrTRG G1–3]	1.29									
C index of final model		0.682 (0.623–0.741)			0.660 (0.605–0.715)			0.701 (0.599–0.803)		

^a^Continuous variable.

MRI; magnetic resonance imaging, OS; overall survival, DFS; disease free survival, LRR; local recurrence rate, VIF; variance inflation factor, HR; hazard ratio, CI; confidence interval, CEA; carcinoembryonic antigen, mr; magnetic resonance imaging before neoadjuvant chemoradiotherapy, T; tumour stage, N; lymph node stage, MRF; mesorectal fascia involvement, EMVI; extramural venous invasion, ymr; magnetic resonance imaging after neoadjuvant chemoradiotherapy, mrTRG; magnetic resonance tumour regression grade, G; grade.

The multivariable analysis of DFS was similar to that of OS. mrEMVI (HR, 2.05; p < 0.001) was the only significant variable among mr findings. Meanwhile, among ymr findings, ymrN (HR, 1.84; p < 0.004), ymrMRF (HR, 1.56; p = 0.054), and ymrEMVI (HR, 1.67; p = 0.026) remained significant. Regarding mr and ymr findings for DFS, Harrell’s C-indices for the final models were 0.631 (95% CI, 0.576–0.686) and 0.660 (95% CI, 0.605–0.715), respectively (Table 3).

There was no significant variable among mr findings for LRR. Among ymr findings, ymrMRF and ymrEMVI remained significant, having HRs of 2.72 (p = 0.013) and 2.42 (p = 0.021), respectively. Regarding mr and ymr findings for LRR, Harrell’s C-indices for the final models were 0.617 (95% CI, 0.529–0.705) and 0.701 (95% CI, 0.599–0.803), respectively (Table 3).

### Survival outcome according to number of ymr risk factors

**Figure 1:**
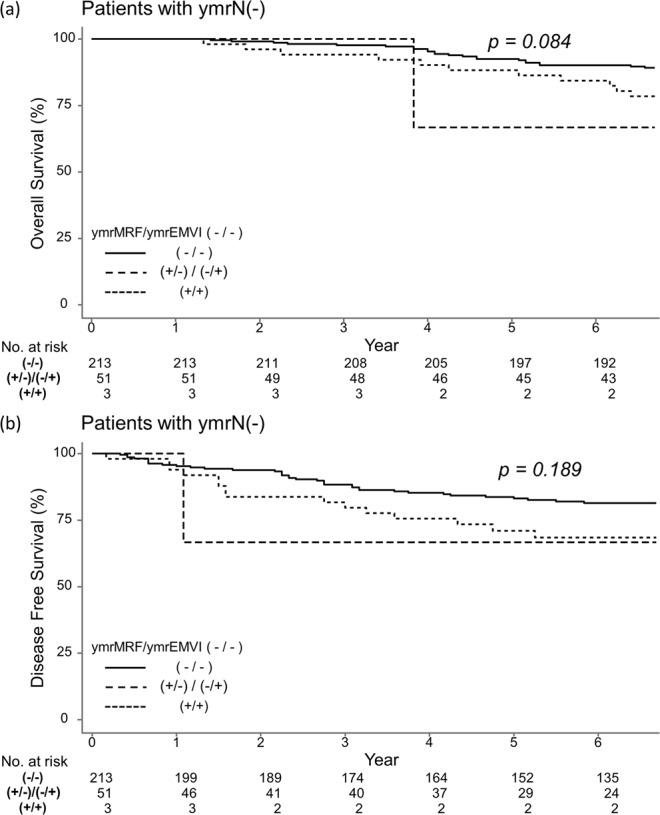
Overall survival (**a**) and disease free survival (**b**) of ymrN(−) patients.

We checked each survival outcome according to the number of ymr risk factors. For DFS and OS, patients were stratified according to ymrN status. Among ymrN(−) patients, only three patients were ymrMRF(+) and ymrEMVI(+), and there was no significant difference between the DFS and OS groups (Fig. 1).

**Figure 2:**
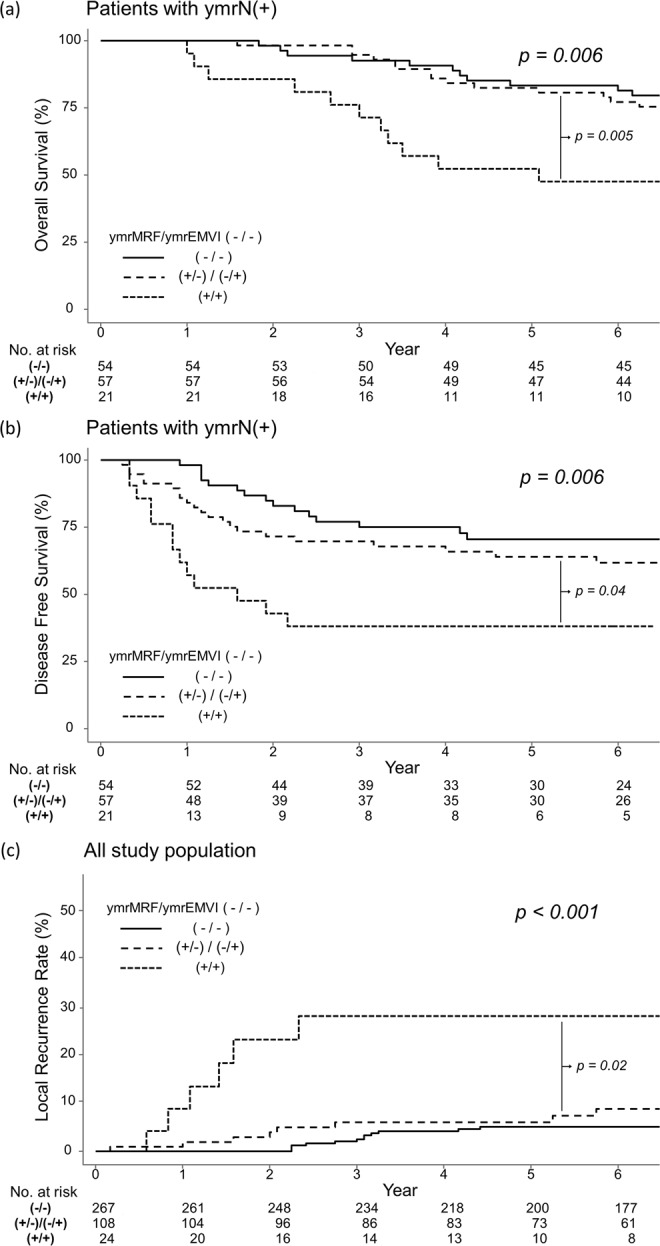
Overall survival (**a**) and disease free survival (**b**) of ymrN(+) patients and local recurrence rate (**c**) of all patients according to ymrMRF and ymrEMVI status.

In the ymrN(+) group, the 5-year OS was significantly worse in ymrMRF(+) and ymrEMVI(+) patients, that is, 52.4% (95% CI, 34.8–78.8), much lower than 83.3% (95% CI, 74.0–93.9) in patients without such findings (p < 0.006) (Fig. 2a). In the same stratum, ymrMRF(+) and ymrEMVI(+) patients also showed poor 5-year DFS of 38.1% (95% CI, 22.1–65.7), compared with 70.5% (95% CI, 58.9–84.3) in patients without such findings (p < 0.006) (Fig. 2b). The 5-year LRR was significantly higher in ymrMRF(+) and ymrEMVI(+) patients, that is, 27.7% (95% CI, 6.1–44.4), higher than 5% (95% CI, 2.2–7.8) in patients without such findings (p < 0.001) (Fig. 2c).

## Discussion

This study revealed that ymr findings tended to be more associated with the patient’s prognosis than mr findings. Among ymr findings, ymrN, ymrMRF, and ymrEMVI were independent prognostic factors for rectal cancer after CRT. When patients had ymrMRF and ymrEMVI, all survival outcomes were found to be significantly worse.

One consistent finding of our study was ymr findings having better prognostic relevance than mr findings. Several studies have demonstrated the prognostic significance of post-CRT MRI findings, and a few of them have shown better clinical value of post-CRT MRI findings than pre-CRT MRI findings. Huh *et al*. found that MRI stages II-III after CRT was a poor prognostic factor for 5-year DFS compared with MRI stage I^17^. In one study, among 92 patients with low rectal cancer with unsafe surgical resection margin on initial MRI, no patient was pathologically CRM(+) when they were assessed as “safe” on MRI after neoadjuvant therapy, while 23.9% of patients were pathologically CRM(+) when they remained to be assessed as “unsafe”^16^. Chand *et al*. showed that ymrEMVI is a significant prognostic factor on multivariable analysis^13^. In another study, the patient’s prognosis when ymrEMVI was positive after CRT was worse than when mrEMVI was positive alone^14^. All these studies have shown results consistent with our results, suggesting that post-CRT MRI findings have more prognostic impact than pre-CRT MRI findings and that ymrN, ymrMRF, and ymrEMVI were independent prognostic factors.

Among mr findings, mrEMVI was the only significant prognostic factor in our analysis (Table 3). The prognostic significance of mrEMVI has been reported in several previous studies. A recent study showed mrEMVI as a significant prognostic factor for OS, metastasis-free survival, and LRR in patients treated with CRT followed by surgery^18^. Cho *et al*. reported that mrEMVI(+) predicts systemic recurrence in patients with rectal cancer even after good response to CRT followed by curative surgery^19^. In a pooled analysis of 269 patients who underwent CRT after neoadjuvant chemotherapy, mrEMVI(+) was the only significant prognostic factor among mr findings for distant progression-free survival^20^. mrEMVI(+) was also the only significant variable among mr findings for DFS on multivariable analysis in a study by Patel *et al*. in patients who underwent neoadjuvant chemotherapy^21^. All these studies showed similar results, suggesting mrEMVI as the most important MRI finding before CRT.

Several trials have attempted to identify patients with high-risk features using MRI findings and apply intensified neoadjuvant therapy for better survival outcomes. The GRECCAR4 trial defined an unfavourable response as a tumour volume shrinkage of <75% on MRI or a ymrMRF(+) finding^3^. The RAPIDO trial enrolled patients with mrT3c/d or mrT4a/b, MRF(+), and EMVI(+) findings^22^. Our study revealed that ymrMRF(+) and ymrEMVI(+) were independent risk features. OS, DFS, and LRR were obviously worse in patients with both of these findings. In such patients, standard treatment might be insufficient and intensified treatment should be considered for better survival outcomes. Our previous study on consolidation chemotherapy for rectal cancer showed only marginal improvement in the down-staging rate that was explained partly due to the dilution effect of low risk patients^23^.[4] For future studies on induction or consolidation treatments, risk stratification based on our study will be helpful to evaluate treatment efficacy.

mrTRG has been also reported as a significant prognostic factor in previous studies^9,24^. However, in our study, mrTRG was not significant on multivariable analysis using ymr findings, although it was significant for all survival outcomes on univariable analysis. Additionally, a recent study reported a low correlation between mrTRG and pathologic TRG^25^. Taken together, these findings suggest that mrTRG is a finding different from pathologic TRG and might not be an independent prognostic factor among other ymr findings. Thus, further study is needed to confirm the prognostic significance of mrTRG.

Our study has some limitations. First, we did not address interobserver disagreement in the analysis. However, mrMRF, ymrMRF, and ymrEMVI were reported to have fair to good interobserver agreement in previous studies^12,26,27^. Moreover, in this study, one experienced radiologist reviewed every MRI finding with consistency. Second, emerging MRI prognostic factors such as diffusion-weighted images and diffusion coefficient values were not considered in the analysis. This was partially due to the absence of corresponding MRI findings in part of the study period. Furthermore, we paid attention to MRI findings based on pathology and included all of them in the analysis. Third, the MRI resolutions were different because 1.5-T and 3.0-T MRI systems were used during the study period. However, one study has shown that both 1.5-T and 3.0-T MRI systems have similar accuracy^28^.

In conclusion, this study found that MRI findings after CRT tended to correlate better with the patient’s prognosis than MRI findings before CRT. mrEMVI(+) was the only significant prognostic factor among mr findings, whereas ymrN(+), ymrMRF(+), and ymrEMVI(+) were independent risk factors among ymr findings. In addition, ymrMRF(+) and ymrEMVI(+) showed an additive effect on the patient’s prognosis. Therefore, MRI findings after CRT could be helpful in distinguishing high-risk patients.

## Methods

Institutional review boards of the National Cancer Centre, Korea reviewed this study and waived the requirement for informed consent on the basis of its retrospective design and minimal risk to the participants (NCC2018-0266). However, method was conducted in accordance with the committee’s approved guidelines to protect patients’ health information.

### Patients

**Figure 3:**
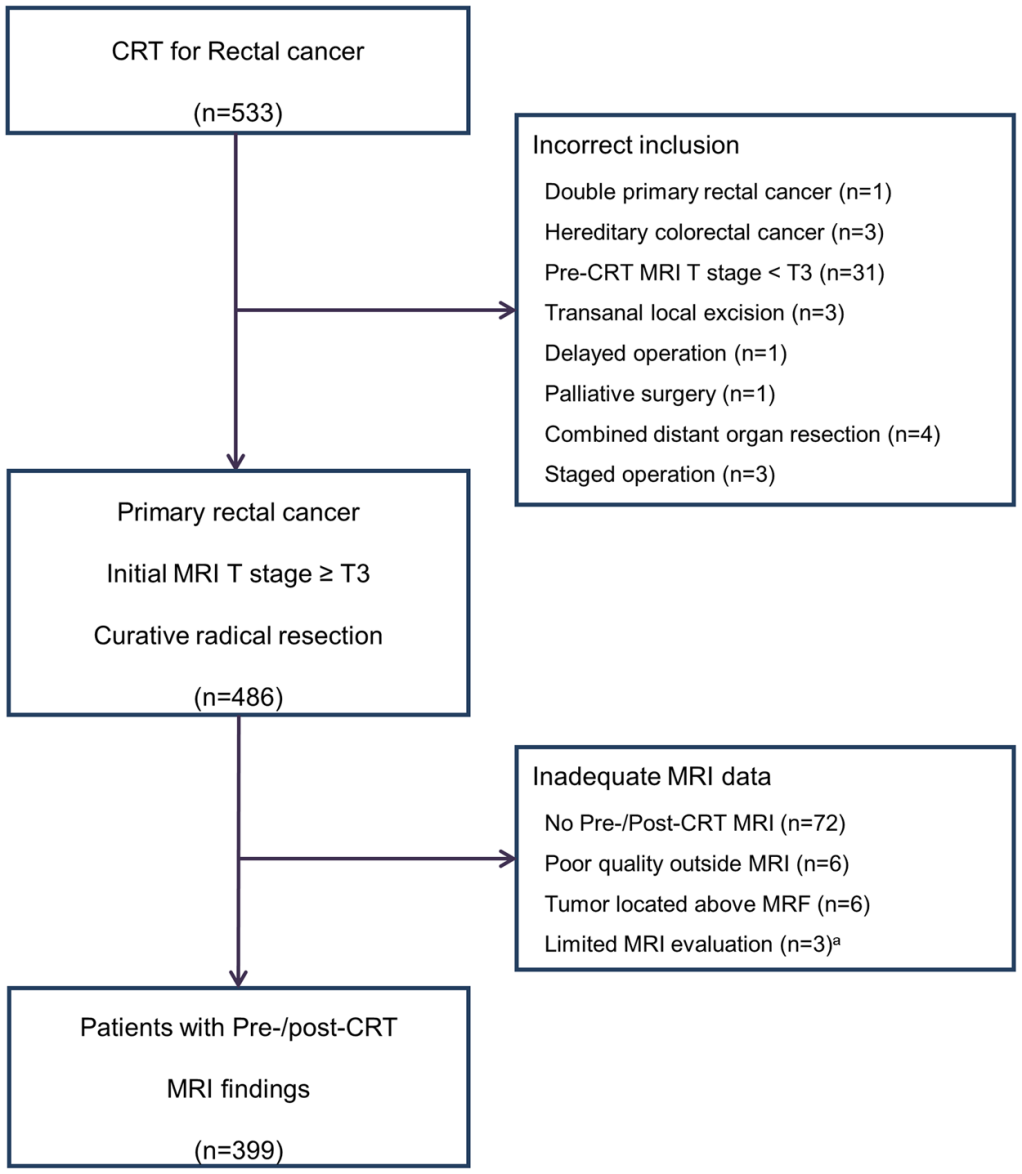
Flow chart showing patient selection. ^a^MRI evaluation was limited due to endoscopic clip. MRI; magnetic resonance imaging, CRT; neoadjuvant chemoradiotherapy.

We reviewed the medical records of 533 patients who underwent CRT for biopsy-confirmed rectal cancer (≤15 cm) from May 2002 to December 2010. The inclusion criteria were as follows: (1) primary rectal cancer without DM or other concomitant malignancy, (2) T3 or T4 classification on initial MRI; (3) adequate mr and ymr data, and (4) curative radical surgery after CRT. Among the 533 patients, 134 were excluded from analysis due to incorrect inclusion (n = 47) and inadequate MRI data (n = 87). The remaining 399 patients were included in the study. Details of the patient selection are summaried in Fig. 3.

### Pre- and post-CRT workups

All patients underwent workups before and after CRT. Rectal MRI with or without rectal ultrasonography was used for local staging. Presence of DM was checked with chest and abdominopelvic computed tomography (CT), and additional positron emission tomography (PET) was performed if necessary. Digital rectal examination (DRE), laboratory tests including carcinoembryonic antigen (CEA), and colonoscopy with biopsy were also checked before CRT. Tumour response was assessed at 6–8 weeks after CRT completion using a protocol similar to pre-CRT workups.

### Rectal MRI assessment

All patients underwent rectal MRI before and after CRT. A phased array body coil (USA Instruments) using the standard imaging protocol was used. One hour before the MRI examination, one bisacodyl suppository (Dulcolax, Boehringer Ingelheim) was administered for bowel preparation. Thirty minutes prior to the MRI, 20 mg of scopolamine butylbromide (Buscopan, Boehringer Ingelheim) was intravenously injected to reduce colonic motility, unless otherwise contraindicated. MR sequences were obtained via the standard T2-weighted, fast spin-echo imaging at three planes (sagittal, axial, and coronal) using the following parameters: echo time (TE)/repetition time (TR) of 80–110/2500–8600 ms, slice thickness of 3–5 mm, a 1-mm intersection gap, echo train length of 16–32, matrix of 224 × 224 to 800 × 538, no fat saturation, and a field of view of 150 × 150 to 360 × 360. Axial, T1-weighted, three-dimensional spoiled-gradient-echo sequence was also performed. 1.5-T MRI (Signa 1.5-T, GE Medical Systems, Boston, MA, USA, n = 505) and 3.0-T MRI (Achieva 3.0 T, Philips Healthcare, Amsterdam, Netherlands, n = 260; Signa HDx 3.0 T, GE Medical Systems, n = 20) systems were used for assessment. One radiologist with 11 years of experience reviewed all rectal MRI scans. The radiologist was blinded from clinical information of patients. All readings of reviewed rectal MRI scans included information on T, N, MRF, EMVI, and mrTRG. N(+) was defined when there was a lymph node with any findings of larger than 5 mm in short diameter, irregular margin or heterogeneous signal intensity. MRF(+) was defined as a tumour, lymph node, EMVI, or tumour deposit located <1 mm from the MRF. Scores of 3 and 4 were considered EMVI(+) using the scoring system presented by Smith *et al*.^11^ mrTRG was assessed based on the grading system presented by Patel *et al*.^9^.

### Treatment and follow-up

A dose of 50.4 Gy was administered for 5.5 weeks as long-course radiotherapy. Neoadjuvant chemotherapy was administered concurrently with radiotherapy. The common chemotherapy regimens were 5-fluorouracil (n = 205) and capecitabine based (n = 109); other regimens included oral tegafur/uracil (n = 53), irinotecan (n = 29), and oxaliplatin based (n = 1) regimens. Two patients underwent radiotherapy alone due to avoidance of chemotherapy.

TME surgery was performed for all patients at 42 days (interquartile range, 37–48) after CRT completion and 2 (interquartile range, 2–4) days after post-CRT MRI assessment. Abdominoperineal resection was performed in 56 (14.0%) patients, and the remaining 343 patients underwent a sphincter-preserving operation. In 14 patients, adjacent pelvic organs, mainly vagina with or without uterus, were resected due to suspicious tumour invasion. All patients were considered for adjuvant chemotherapy regardless of the postoperative histologic findings. Adjuvant chemotherapy commenced at 3–6 weeks after surgery in 381 (95.5%) patients. Fluoropyrimidine (n = 309), tegafur/uracil (n = 37), oxaliplatin (n = 33), and irinotecan based (n = 2) regimens were used for adjuvant chemotherapy.

All patients were instructed to visit the hospital every 3 months for the first 2 years, every 6 months for the next 3 years, and every year thereafter. DRE, laboratory tests including CEA, and chest X-ray were performed at every visit. Abdominopelvic CT with or without chest CT was taken every 6 months, and colonoscopy was performed at 1, 3, and 5 years after surgery. The follow-up duration was shortened if there was any sign of recurrence, and rectal MRI or PET was added if necessary. Recurrence was confirmed by biopsy or radiologic findings on serial imaging. Tumour recurrence within the pelvis was considered as local recurrence, whereas that outside the pelvis was defined as DM.

### Statistical analysis

The Kaplan-Meier method and log-rank test were used to evaluate survival outcomes. The survival time was calculated from the date of surgery to the first event or date of last follow-up for censored data. The events for each survival outcome were defined as follows: death from any cause for overall survival (OS); any recurrence, second primary colorectal or other malignancy, or death from any cause for DFS; and recurrence within the pelvis for LRR.

Univariable analyses of MRI findings were performed using Cox regression for OS, DFS, and LRR. Age, sex, tumour location, and CEA were included as clinical variables. Subsequent stepwise multivariable Cox regression was performed to derive two final models for OS, DFS, and LRR using the mr and ymr findings, separately. Backward elimination method was applied, and among clinical and MRI variables, only significant variables (p < 0.05) were included for making final predictive models in the univariable analysis. Harrell’s C-index was used as a measure of predictive accuracy for the final model determining the prognostic value of the mr and ymr findings. Statistical analyses were performed using the R Project for Statistical Computing software version 3.2.3 (R Development Core Team, Vienna, Austria).

The datasets generated during and/or analysed during the current study are available from the corresponding author on reasonable request.

## Supplementary information

Supplementary table1 and 2
