# Supplementary material for: Differences in prognostic relevance of rectal magnetic resonance imaging findings before and after neoadjuvant chemoradiotherapy
Source: Sci Rep. 2019 Jul 11;9:10059. doi: 10.1038/s41598-019-46499-9 (PMC6624197; doi:10.1038/s41598-019-46499-9)
Supplement: Supplementary file 1 — Supplementary table1 and 2 [file 41598_2019_46499_MOESM1_ESM.docx]

**Differences in prognostic relevance of rectal magnetic resonance imaging findings before and after neoadjuvant chemoradiotherapy**

Kwang-Seop Song^1^, Dong Woon Lee^1^, Bun Kim^1^, Bo Yun Hur^2^, Min Jung Kim^3^, Min Ju Kim^4^, Chang Won Hong^1^, Sung Chan Park^1^, Hyoung Chul Park^1^, Dae Kyung Sohn^1^, Byung Chang Kim^1^, Kyung Su Han^1^, Jae Hwan Oh^1*^

^1^Research Institute and Hospital, National Cancer Centre, Centre for Colorectal Cancer, Goyang, 10408, Korea

^2^Seoul National University College of Medicine, Healthcare System Gangnam Centre, Department of Radiology, Seoul, 06236, Korea

^3^Seoul National University College of Medicine, Seoul National University Hospital, Department of Surgery, Seoul, 03080, Korea

^4^ Research Institute and Hospital, National Cancer Centre, Department of Radiology, Goyang, 10408, Korea

Correspondence: Jae Hwan Oh, MD, PhD

Centre for Colorectal Cancer, Research Institute and Hospital, National Cancer Centre, 323 Ilsan-ro, lsandong-gu, Goyang, Gyeonggi 410-769, Republic of Korea

Tel: +82-31-920-1637

Fax: +82-31-920-1148

E-mail: [jayoh@ncc.re.kr](mailto:jayoh@ncc.re.kr)

**Supplementary Table 1.** Univariable analysis of MRI variables according to downstaging

| Variable [reference] | OS (event=102) | | | | DFS (event=116) | | | | LRR (event=29) | | |
| --- | --- | --- | --- | --- | --- | --- | --- | --- | --- | --- | --- |
|  | HR | 95% CI | P | HR | | 95% CI | P | HR | | 95% CI | p |
| mrT3-4 → ymrT0-2 | 1 | (Reference) |  | 1 | | (Reference) |  | 1 | | (Reference) |  |
| mrT3-4 → ymrT3-4 | 1.52 | 0.97-2.37 | 0.067 | 1.74 | | 1.13-2.66 | 0.011 | 1.63 | | 0.70-3.82 | 0.258 |
| mrN(-) → ymrN(-) | 1 | (Reference) |  | 1 | | (Reference) |  | 1 | | (Reference) |  |
| mrN(+) → ymrN(-) | 0.91 | 0.53-1.58 | 0.737 | 1.01 | | 0.59-1.74 | 0.966 | 1.87 | | 0.53-6.62 | 0.333 |
| mrN(+) → ymrN(+) | 2.03 | 1.20-3.42 | 0.008 | 2.28 | | 1.35-3.84 | 0.002 | 3.44 | | 0.99-11.97 | 0.053 |
| mrMRF(-) → ymrMRF(-) | 1 | (Reference) |  | 1 | | (Reference) |  | 1 | | (Reference) |  |
| mrMRF(+) → ymrMRF(-) | 0.57 | 0.25-1.32 | 0.189 | 0.90 | | 0.47-1.74 | 0.755 | 0.76 | | 0.18-3.26 | 0.707 |
| mrMRF(+) → ymrMRF(+) | 1.74 | 1.08-2.81 | 0.022 | 1.95 | | 1.25-3.03 | 0.003 | 2.78 | | 1.25-6.19 | 0.012 |
| mrEMVI(-) → ymrEMVI(-) | 1 | (Reference) |  | 1 | | (Reference) |  | 1 | | (Reference) |  |
| mrEMVI(+) → ymrEMVI(-) | 0.93 | 0.55-1.59 | 0.801 | 1.30 | | 0.81-2.08 | 0.280 | 0.92 | | 0.32-2.62 | 0.878 |
| mrEMVI(+) → ymrEMVI(+) | 2.37 | 1.53-3.67 | 0.000 | 2.48 | | 1.63-3.79 | 0.000 | 2.66 | | 1.19-5.94 | 0.017 |

Those MRI variables were not included in making the final model.

MRI; magnetic resonance imaging, OS; overall survival, DFS; disease free survival, LRR; local recurrence rate, HR; hazard ratio, CI; confidence interval, mr; magnetic resonance imaging before neoadjuvant chemoradiotherapy, ymr; magnetic resonance imaging after neoadjuvant chemoradiotherapy, T; tumour stage, N; lymph node stage, MRF; mesorectal fascia involvement, EMVI; extramural venous invasion

**Supplementary Table 2.** Validity and reliability assessment between MR findings and pathology

1. ymrT

|  | ypT0-1 | ypT2 | ypT3 | ypT4 |
| --- | --- | --- | --- | --- |
| ymrT0-1 | 16 | 5 | 1 | 0 |
| ymrT2 | 35 | 55 | 17 | 0 |
| ymrT3 | 17 | 37 | 182 | 3 |
| ymrT4 | 1 | 1 | 12 | 17 |
| Validity (95% CI) | Weighted Kappa= 0.53 (0.46-0.60) | | | |

1. ymrN

|  | ypN(-) | ypN(+) | Validity (95% CI) |
| --- | --- | --- | --- |
| ymrN(-) | 226 | 41 | Sensitivity: 0.71 (0.63-0.78)  Specificity: 0.88 (0.83-0.92)  Accuracy: 0.82 (0.78-0.86) |
| ymrN(+) | 31 | 101 |  |

1. ymrMRF

|  | ypCRM(-) | ypCRM(+) | Validity (95% CI) |
| --- | --- | --- | --- |
| ymrMRF(-) | 336 | 4 | Sensitivity: 0.86 (0.67-0.96)  Specificity: 0.91 (0.87-0.93)  Accuracy: 0.90 (0.87-0.93) |
| ymrMRF(+) | 35 | 24 |  |

Concerning EMVI, correlation was not possible because there was no detailed pathologic data on whether EMVI was of intramural or extramural type during most of the involved study period.

ymr; magnetic resonance imaging after neoadjuvant chemoradiotherapy, T; tumour stage, N; lymph node stage, MRF; mesorectal fascia involvement, CRM; circumferential resection margin, EMVI; extramural venous invasion, CI; confidence interval
